# Probabilistic Coverage Constraint Task Assignment with Privacy Protection in Vehicular Crowdsensing

**DOI:** 10.3390/s23187798

**Published:** 2023-09-11

**Authors:** Zhe Li, Xiaolong Liu, Yang Huang, Honglong Chen

**Affiliations:** College of Control Science and Engineering, China University of Petroleum (East China), Qingdao 266580, China; lizhe@upc.edu.cn (Z.L.); lxl18970691736@163.com (X.L.); yanghuang97@outlook.com (Y.H.)

**Keywords:** vehicular crowdsensing, spatio-temporal correlation, task assignment, differential privacy

## Abstract

The increasing popularity of portable smart devices has led to the emergence of vehicular crowdsensing as a novel approach for real-time sensing and environmental data collection, garnering significant attention across various domains. Within vehicular crowdsensing, task assignment stands as a fundamental research challenge. As the number of vehicle users and perceived tasks grows, the design of efficient task assignment schemes becomes crucial. However, existing research solely focuses on task deadlines, neglecting the importance of task duration. Additionally, the majority of privacy protection mechanisms in the current task assignment process emphasize safeguarding user location information but overlook the protection of user-perceived duration. This lack of protection exposes users to potential time-aware inference attacks, enabling attackers to deduce user schedules and device information. To address these issues in opportunistic task assignment for vehicular crowdsensing, this paper presents the minimum number of participants required under the constraint of probability coverage and proposes the User-Based Task Assignment (UBTA) mechanism, which selects the smallest set of participants to minimize the payment cost while measuring the probability of accomplishing perceived tasks by user combinations. To ensure privacy protection during opportunistic task assignment, a privacy protection method based on differential privacy is introduced. This method fuzzifies the sensing duration of vehicle users and calculates the probability of vehicle users completing sensing tasks, thus avoiding the exposure of users’ sensitive data while effectively assigning tasks. The efficacy of the proposed algorithm is demonstrated through theoretical analysis and a comprehensive set of simulation experiments.

## 1. Introduction

In recent years, the widespread adoption of portable smart IoT devices has become an integral part of people’s lives [1,2]. These devices have evolved with the rapid advancement of science and technology, now equipped with a diverse array of powerful embedded sensors, including cameras, microphones, GPS, gyroscopes, accelerometers, and compasses. Together, these sensors collaboratively gather extensive information about human activities and the surrounding environment. The convergence of mobile smart terminals, featuring high-performance built-in sensors, and the rapid growth of the Internet have given rise to vehicular crowdsensing (MCS) [3,4]. This paradigm offers a novel approach for real-time sensing and environmental data collection. Vehicular crowdsensing effectively utilizes the mobile population to enable low-cost, real-time, large-scale, and fine-grained data collection. It transforms vehicle users from passive data consumers into active data providers, thereby introducing a new service mode for vast multi-source heterogeneous data awareness in the realm of the large-scale Internet of Things [5,6,7]. The great potential of vehicular crowdsensing has led to its widespread application across various fields, including environmental monitoring, smart cities, industrial sensors, city awareness, and social networks [8,9,10]. Its ability to harness the collective power of mobile users to contribute to data collection has made it instrumental in addressing diverse challenges and needs in these domains.

Task assignment stands as a critical research aspect in the realm of vehicular crowdsensing [11,12,13,14,15]. The success of vehicular crowdsensing largely hinges on the design of effective task assignment schemes. As the number of vehicle users and perceived tasks in the Cluster Awareness System continues to grow rapidly, an efficient task allocation mechanism becomes crucial to enhance user participation and achieve substantial benefits. Recently, numerous researchers have devoted efforts to developing task allocation mechanisms. Some of these works [16,17] focus on maximizing perceived quality or minimizing incentive costs under various constraints, while some other works aim to reduce energy consumption [18]. Additionally, there has been significant research attention directed toward the assignment of location-dependent and time-sensitive tasks [19]. These endeavors seek to address the challenges posed by task complexity and dynamic environmental conditions, further advancing the capabilities of vehicular crowdsensing systems.

Most of the existing research [20,21,22] takes into account the effective time of perceived tasks, which can be categorized as either time-sensitive tasks or delay-tolerant tasks. Time-sensitive tasks [23] require immediate completion and demand high time-effectiveness, typically due to emergency situations. On the other hand, delay-tolerant tasks [24] target relatively stable perceptual activities and can be completed over an extended period. Some delay-tolerant task assignment schemes focus on recommending appropriate routes for vehicle users or scheduling tasks based on user interests and habits. However, the aforementioned studies primarily focus on task deadlines while disregarding the time required to perform each task. This oversight is particularly critical when the available time of each vehicle user is limited, as it significantly impacts the global optimization of task assignment. Thus, there is an urgent need to design an effective task assignment scheme that considers the sensing duration of vehicle users in a spatio-temporal cluster-aware system.

Privacy protection concerns in task assignment also hold significant importance [25,26,27]. Vehicular crowdsensing faces potential security threats due to the large volume of data streams and the absence of robust security mechanisms. The interactions between vehicle users, platforms, and third parties create a scenario wherein privacy breaches become a serious issue. In vehicular crowdsensing, users are required to upload their perceived data to the platform, which can contain sensitive information such as user identities, locations, and other private attributes. If the platform experiences a security breach or loses trustworthiness (e.g., by selling data to third parties for profit), users’ privacy will be exposed to unauthorized entities.

Recently, researchers have proposed several methods to address user privacy protection in vehicular crowdsensing. To et al. [28] introduced a mechanism based on differential privacy and geo-broadcasting to safeguard users’ location privacy. The approach confuses the number of users in a particular area using a trusted third-party platform, thus preventing attackers from inferring users’ precise locations. Similarly, Wang et al. [29] explored the issue of publishing statistical information for location-based datasets. They employed differential privacy technology to ensure that users’ personal location information remains uncompromised. However, the privacy protection schemes currently available are not applicable to vehicular crowdsensing scenarios that prioritize perception duration. Currently, there is no research focusing on privacy protection concerning perception duration in task assignment. Consequently, there is an urgent need to develop effective solutions that cater to users’ privacy protection requirements while optimizing task assignment in space–time-related vehicular crowdsensing. These solutions aim to prevent users’ sensitive information from being disclosed to unauthorized entities.

To sum up, this paper focuses on the space–time characteristics of vehicular crowdsensing and conducts research on task allocation in this context. Additionally, it designs a privacy protection mechanism to safeguard vehicle users’ sensitive data while ensuring efficient task allocation. The research encompasses two main areas: probabilistic task assignment with a probabilistic coverage constraint in vehicular crowdsensing, and privacy protection in the task assignment process. By addressing these aspects, this study successfully resolves the issues of ineffective task assignment caused by neglecting the spatio-temporal characteristics of users and tasks. Moreover, it tackles the problem of user-sensitive data leakage, resulting from the oversight of privacy protection concerning users’ perceptual time in the task assignment process of spatio-temporal-related vehicular crowdsensing.

## 2. Related Work

### 2.1. Task Assignment without Privacy Protection

Task assignment stands as a crucial research aspect in vehicular crowdsensing. It involves the rational allocation of perceived tasks to vehicle users by servers, considering system optimization objectives and real-world constraints. And the research delves into the trade-off between cost and benefit, representing two opposing factors. Li et al. [30] considered the problem of dynamic participant selection with heterogeneous sensing tasks, that is, the sensing tasks come in real time and have different requirements for space–time coverage. Its goal is to minimize the sensing cost while maintaining a certain probability coverage level. Wang et al. [31] proposed a two-stage hybrid task assignment framework that can be used to recruit both opportunistic and participatory users, with the goal of maximizing the number of tasks completed under the constraints of the overall budget. Wang et al. [32] researched the robust task allocation problem in MCS systems, aiming to enhance the robustness of the allocation scheme and minimize the users’ detour costs.

However, the majority of existing research only focuses on the deadline of the perceptual task, disregarding the working time of the vehicle user during task execution. Furthermore, few studies take into account both the space–time constraints of vehicle users and perceived tasks. In a cluster-aware system, vehicle users typically possess different route budgets and time budgets. Additionally, sensory tasks are linked to specific locations and sensing duration. As the complexity and scale of perception systems increase, the corresponding optimization problems for task assignment is NP-hard, making it challenging to find effective optimal algorithms. In such cases, suboptimal algorithms need to be considered. Hence, given the unique characteristics of task perception duration and the space–time constraints of users and tasks in spatio-temporal correlated vehicular crowdsensing, there is a critical need to thoroughly investigate the spatio-temporal-related task assignment problem. The objective is to achieve optimal task assignment while considering the intricate real-world space–time constraints. In addition, none of the above works take into account the protection of user privacy, which will affect the effectiveness of task assignment.

### 2.2. Task Assignment with Privacy Protection

In the task assignment process of vehicular crowdsensing, vehicle users often share data with the platform through wireless access points or cellular infrastructure, which involves many sensitive data (such as location information, device ID, etc.). These sensitive data may leak users’ daily travel or real identity, resulting in a significant reduction in users’ motivation to participate in perceived tasks. In recent years, some research work has focused on the issue of privacy protection in the process of task assignment for vehicular crowdsensing. Zhang et al. [33] assumed that, in the absence of any trusted entity, users were allowed to locally confuse their location using a method based on differential privacy to reduce the risk of location privacy disclosure. This method about trusted entity [34,35] could ensure that opponents with prior knowledge could only obtain little additional information from the ambiguous location. Wang et al. [36] proposed a task assignment framework for location privacy protection with geographic location confusion to minimize the expected travel distance of selected vehicle users under the constraints of differential privacy and distorted privacy. Xu et al. [26] proposed a privacy-preserving fine-grained task assignment scheme for the crowdsensing system and designed a novel incentive mechanism based on the capabilities of workers. To be specific, a ciphertext-policy attribute-based encryption (CP-ABE) scheme with the characteristics of hidden policy is adopted to select workers and protect the privacy. Qian et al. [37] proposed an optimal location privacy preserving and service quality guaranteed task allocation in vehicle-based crowdsensing networks and utilized differential privacy to preserve participants’ location privacy, where every participant can submit the obfuscated location to the platform instead of the real one.

The current research on privacy protection during the task assignment process for vehicular crowdsensing is still in its early stages. While some mechanisms have been proposed, they suffer from issues like high computational costs, vulnerability, or reliance on trusted third-party platforms. Moreover, the existing privacy mechanisms in task assignment predominantly focus on safeguarding user location information, overlooking the protection of user perceived time. However, it is crucial to consider the privacy of user-perceived time in space–time-related cluster awareness systems since attackers can potentially infer user schedules and device information through these data. Therefore, there is a pressing need to delve deeper into privacy protection issues in the task assignment process for space–time-related vehicular crowdsensing. This research aims to achieve optimal task assignment while ensuring the fulfillment of users’ privacy protection requirements.

## 3. Problem Description

In this paper, we mainly focus on the spatio-temporal-related vehicular crowdsensing, in which the perception task is not only located in a specific spatial region, but also needs a certain amount of sensing time to complete. In this model, vehicular crowdsensing aims to recruit vehicle users who can dedicate a specific duration to perform tasks effectively. For instance, in application scenarios like crowd monitoring, air quality assessment, and noise level detection, the perception tasks necessitate vehicle users to collect data (such as congestion levels, air pollution indicators, and noise levels) within a certain time frame to obtain sufficient and meaningful information.

The environmental department of the municipal government has planned an air quality monitoring project to update the air quality index every hour throughout the day. However, due to the high costs associated with deploying, maintaining, and consuming energy for basic monitoring equipment, the municipal government decided to leverage vehicular crowdsensing to accomplish the task. The project aims to recruit vehicle users equipped with smartphones, who will install a specific application to perceive air quality. To execute the project effectively, the sensing area is divided into 45 regions based on signal tower coverage. Each day is further divided into 10 sensing cycles, spanning from 8:00 to 18:00, with each cycle lasting for 1 h. About 1000 smartphone users have agreed to participate in the two-week air quality sensing project. Each selected vehicle user is responsible for sensing air quality in a specific area and transmitting the collected data through the designated signal tower. The project’s objective is to ensure that the tasks performed by the recruited users cover at least 90% of the 45 areas in all sensing cycles. Consequently, in each sensing cycle, no fewer than 41 air quality monitoring tasks should be completed. To minimize the total project cost while ensuring the perceived quality, the municipal government intends to select the minimum number of users from the pool of 1000 candidates. For this purpose, the municipal government obtained the two-week call records of the 1000 candidates (with time stamps of the calls and communication tower IDs, after desensitizing other information) with the assistance of telecom operators, after obtaining the consent of users.

**Figure 1:**
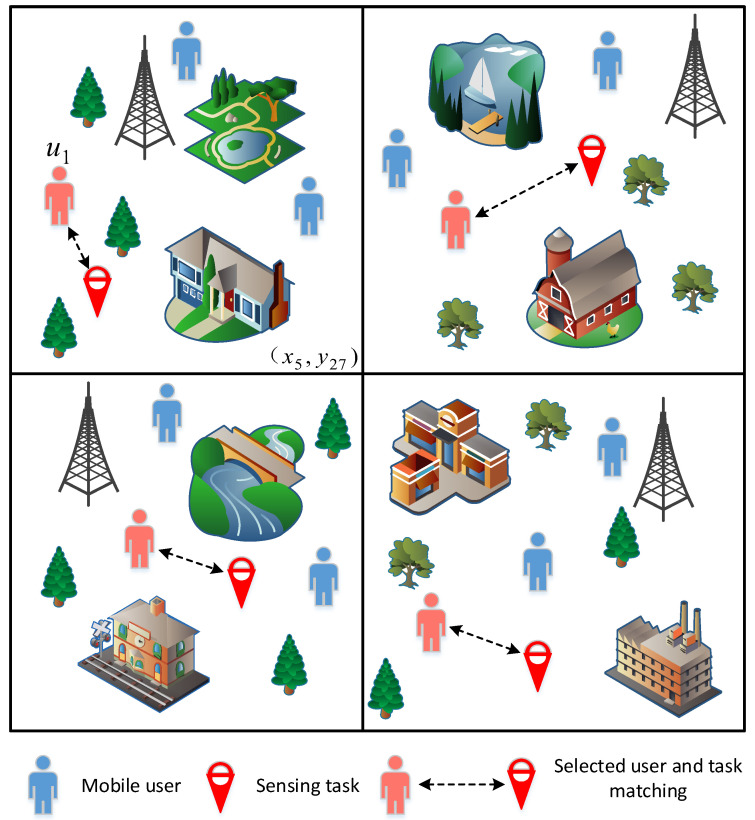
An example of task assignment.

As shown in Figure 1, for example, vehicle user $u_{1}$ is assigned a sensing task associated with the space-time unit $\left(x_{5},y_{27}\right)$ (that is, the sensing task is in the fifth sensing cycle and located in the 27th perception area), user $u_{1}$ air quality monitoring needs to be carried out in the space–time unit $\left(x_{5},y_{27}\right)$. Air quality monitoring is a time-dependent task in vehicular crowdsensing, requiring vehicle users to spend a specific duration for obtaining effective data. To ensure the successful completion of the sensing task, users are required to upload their stay time in the sensing area during the designated sensing cycle. To protect users’ privacy, the stay time data will be subject to privacy protection measures. Upon selecting the smallest vehicle user set based on users’ historical calls and mobile records, each chosen participant will receive a fixed reward and activate the application on their mobile phones. Throughout the entire project, when vehicle users reach a specific sensing area within the sensing cycle, they can utilize the application on their mobile phones to perceive and upload air quality data.

## 4. System Model

As can be seen from the above examples, the goal of this research work is to select the smallest set of participants for the perception task, and ensure that in each sensing cycle, there is a sensing area that is not lower than the predefined coverage. We propose a vehicular crowdsensing opportunistic task allocation problem. The main goal is to minimize the total incentive cost while satisfying the predefined coverage constraints. In the described model, the project can exist for a period of time (for example, two weeks), including multiple sensing cycles, such as 10 cycles per day, from 8:00 to 18:00, each cycle of 1 h. We set that, if a vehicle user completes the sensing task in the sensing period *i* and the sensing area *a*, it is said that the area a is covered in the cycle *i*. If a vehicle user goes to multiple sensing areas and completes the sensing tasks in these areas in the sensing cycle *i*, it is said that these sensing areas are covered in the sensing cycle *i*. Therefore, the goal of this study is to give a group of call records and confused residence time data of vehicle users, select the minimum number of users, and meet the space–time coverage constraint of the specified proportion, that is, in all sensing cycles, the proportion of the covered sensing area should be equal to or greater than the set coverage threshold.

**Figure 2:**
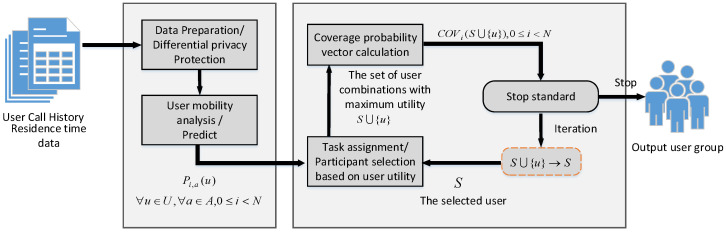
The task assignment framework.

According to the above definitions, we model the problem as follows. Given a group of vehicle users U willing to participate in the sensing project, the divided sensing area set *A*, the call records (including call timestamp and communication tower ID), and dwell time data of all vehicle users, we define S as the participants (S⊂U) selected from the vehicle user set U, $a_{i}\left(S\right)$ is the set of sensing areas covered by the participant set S in the I sensing cycle, then the whole problem is to find a subset S of the vehicle user set U, which can be expressed as follows:

(1) $$\begin{array}{c} \mathrm{minimize}|S|,\\ \;s.t.\;\left\{\begin{array}{c} \frac{\left|a_{i}\left(S\right)\right|}{\left|A\right|}\geq R\\ 0\leq i\leq N \end{array}\right. \end{array}$$

where *R* is the preset coverage threshold and N is the number of sensing cycles. Due to the fact that we cannot know in advance when vehicle users will appear and in which sensing area to perform sensing tasks (that is, when selecting the set of participants, $a_{i}\left(S\right)$ is uncertain), the whole task allocation problem can be divided into two sub problems: vehicle user mobility prediction problem and prediction-based participant selection problem. The swarm intelligence sensing project uses a platform-centric task assignment method. The platform collects and stores the historical call records and residence time data of vehicle users. Before the sensing task is executed, participants are selected from all candidate vehicle users. Only the selected participants are required to perform the task and upload the perceived results in each sensing cycle. Considering the two sub problems of task assignment, the mechanism we designed is divided into two stages. In the first stage, we predict the flow of users between perceived areas based on the historical call records and residence time data of vehicle users, and in the second stage, we select participants according to the prediction results. The overall system framework is shown in Figure 2.

### 4.1. The First Stage—Data Preparation and User Mobility Analysis

After obtaining the call records and residence time data of all candidate vehicle users, the vehicular crowdsensing platform proceeds to calculate the mobility of each user. This mobility metric represents the probability of each user successfully completing the sensing task in a specific sensing area during a designated sensing cycle. The calculation process involves two steps as follows:

(1) Calculate the probability that each user makes at least one call in a specific sensing cycle and sensing area. First, map the historical call record of each user to *n* sensing cycles, so that the parameter $\lambda_{u,I,a}$ can be calculated, that is, the average number of calls made by vehicle user u(u∈U) in sensing area a(a∈A) in sensing cycle i(0≤i<N). Get $\lambda_{u,I,a}$, the probability $P_{i,a}^{\prime }\left(u\right)$ that the vehicle user u makes at least one call in the sensing period *i* and the sensing area *a* can be calculated.

(2) Calculate the probability that each user’s residence time in a specific sensing cycle and sensing area is equal to or greater than the sensing duration required by the task. What the vehicle user uploads is the residence time after confusion, and the sensing tasks concerned in this paper are all time-dependent tasks (that is, the task needs a certain perception time to be completed, and the user can obtain valid data). Therefore, it is necessary to calculate the probability $P_{i,a}^{\prime \prime }\left(u\right)$ that the residence time of the vehicle user *u* in the sensing cycle *i* and sensing area *a* is not less than the sensing duration required by the task. Finally, the probability $P_{i,a}\left(u\right)$ of each user completing the task in a specific sensing cycle and area is the product of $P_{i,a}^{\prime }\left(u\right)$ and $P_{i,a}^{\prime \prime }\left(u\right)$.

### 4.2. The Second Stage—Participant Selection

According to the user mobility data obtained in the first stage, we propose a user utility-based task assignment mechanism (UBTA), which iteratively selects participants by calculating the utility of the user portfolio, which is divided into three steps:

(1) Select the most effective user among all candidate vehicle users, and add the user to the recruited user set.

(2) Among the remaining non-recruited vehicle users, select the user who has the greatest utility after combining with the recruited vehicle users, and add this user to the recruited user set.

(3) Continue to select and add new vehicle users until the recruited user set can cover the sensing area of the preset threshold in each sensing cycle.

## 5. Task Assignment Algorithm Based on User Utility

In this section, users utilize the Laplacian mechanism to add noise to their own residence time, and then upload the confusing residence time instead of the real residence time to the platform to protect their privacy. In this process, only the platform and the users are aware of the privacy protection mechanism, so the attackers cannot infer from the residence time which user completed the task. At the same time, based on the fact that the platform is not interfered with by confusing data, the User6Based Task Allocation (UBTA) algorithm that we propose selects the smallest set of participants to minimize the payoff cost while measuring the probability of performing the tasks perceived by the user combinations. Moreover, the Poisson distribution, commonly used in queuing theory, is employed in this research. In real-life scenarios, numerous situations adhere to the Poisson distribution, such as passengers arriving at an airport or calls received by a customer service desk. Similarly, the Poisson distribution finds relevance in the domain of cellular networks. For this study, we assume that the call sequence adheres to a non-homogeneous Poisson process. Consequently, we model the probability of each vehicle user completing each sensing task based on the Poisson distribution. In the sensing period i(0≤i<N), the probability that the vehicle user *u* makes *n* calls in the sensing area a(a∈A) can be expressed as follows:

(2) $$p_{i,a}\left(u,n\right)=\lambda_{u,i,a}^{n}*e^{-\lambda_{u,i,a}}/n!,$$

where $\lambda_{u,i,a}$ represents Poisson strength, and its estimated value is the average number of calls made by vehicle user *u* in sensing area *a* in sensing period *i*. For example, in order to estimate $\lambda_{u,i,a}$ of sensing period *i* from 08:00 to 09:00, we will calculate the average number of calls made by vehicle user *u* in sensing area *a* from 08:00 to 09:00 in historical call records. Therefore, the probability that the vehicle user *u* makes at least one call in the sensing area *a* during the sensing period *i* can be calculated as follows:

(3) $$P_{i,a}^{\prime }\left(u\right)=\sum_{n=1}^{+\infty }p_{i,a}\left(u,n\right)=1-e^{-\lambda_{u,i,a}},\;$$

In this paper, the sensing time of vehicle users plays a crucial role in determining whether a task can be successfully completed. The sensing task is considered effectively completed only when the vehicle users reach a specific perception area and remain there for a duration equal to or exceeding the required perception time. To facilitate accurate and efficient task assignment, vehicle users can submit their stay time in the sensing period and area to the platform. However, privacy concerns arise due to potential malicious attackers who may infer daily habits, tracks, and other sensitive information from the real stay time, leading to privacy risks. To address this issue, this paper employs the Laplacian mechanism, which locally confuses the residence time. Subsequently, vehicle users upload the fuzzy residence time instead of the actual residence time to the platform, thereby safeguarding their privacy. We use $l_{u,i,a}$ to represent the residence time of the vehicle user *u* in the perception area *a* during the sensing period *i*, and ${\tilde{l}}_{u,i,a}$ to represent the confusing residence time generated by adding Laplace noise to the real residence time. When $l_{u,i,a}$ is known, it is easy to know whether the vehicle users have the ability to complete the sensing task or not. However, the existing methods cannot predict the user mobility problem because the users only upload the confused staying time, so a new solution is urgently needed.

Considering the noise added to the residence time, we transform the problem into the probability that the fuzzy residence time is greater than the perceived duration. When the sensing duration required to define the perception task is δ, the goal of the platform is to obtain the probability that $l_{u,i,a}$ is not less than δ, which is expressed as $P(l_{u,i,a}\geq \delta )$. Assume that vehicle user U adds Laplace noise to the real residence time $l_{u,i,a}$ by using privacy budget parameter ϵ, and obtains the fuzzy residence time ${\tilde{l}}_{u,i,a}$ and uploads it to the platform. Therefore, we can obtain:

(4) $$l_{u,i,a}={\tilde{l}}_{u,i,a}-\eta ,\;\eta ∼Laplace(0,\;1/\epsilon ),$$

where η is a variable that obeys Laplace distribution, and the smaller the privacy budget parameter ϵ, the greater the added noise and the higher the level of privacy protection. For $P(l_{u,i,a}\geq \delta )$, there is the following equation:

(5) $$\begin{array}{cc} P\left(l_{u,i,a}\geq \delta \right) & =P\left({\tilde{l}}_{u,i,a}-\eta \geq \delta \right) \end{array}$$

(6) $$\begin{array}{cc} \; & =P\left({\tilde{l}}_{u,i,a}-\delta \geq \eta \right) \end{array}$$

(7) $$\begin{array}{cc} \; & =P\left(\eta \leq {\tilde{l}}_{u,i,a}-\delta \right), \end{array}$$

where ${\tilde{L}}_{u,I,a}-\delta$ platform can be calculated, the above equation can be regarded as the probability problem of continuous random variables; then, we can obtain the following equation:

(8) $$P\left(\eta \leq {\tilde{l}}_{u,i,a}-\delta \right)=\int_{-\infty }^{{\tilde{l}}_{u,i,a}-\delta }f\left(\eta \right)d\eta ,$$

where $f\left(\eta \right)$ represents the probability density function of variable η:

(9) $$f\left(\eta \right)=\frac{\epsilon }{2}e^{-\epsilon \left|\eta \right|}.$$

As a result, the platform can calculate the probability $P_{i,a}^{\prime \prime }\left(u\right)$ of vehicle user *u* in perception cycle *i* and perception region *a* not less than the sensing duration required by the task according to the fuzzy residence time ${\tilde{L}}_{u,I,a}$ and privacy budget parameter ϵ.

(10) $$P_{i,a}^{\prime \prime }\left(u\right)=P\left(\eta \leq {\tilde{l}}_{u,i,a}-\delta \right)=\int_{-\infty }^{{\tilde{l}}_{u,i,a}-\delta }\frac{\epsilon }{2}e^{-\epsilon \left|\eta \right|}d\eta .$$

Based on the above calculation process, we can obtain the probability $P_{i,a}\left(u\right)$ of each user completing the sensing task in specific sensing cycle *i* and sensing area *a*, that is, the probability that sensing area *a* is covered by vehicle user *u* in sensing cycle *i*. In addition, $P_{i,a}\left(u\right)$ is the product of $P_{i,a}^{\prime }\left(u\right)$ and $P_{i,a}^{\prime \prime }\left(u\right)$.

Next, we calculate the utility of users. Given all candidate mobile user set U and selected participant set S, first combine each unselected user (∀U∈U∖S) with the selected participant to generate a combinatorial set $S\cup \left\{u\right\}$. Then, calculate the utility of each combinatorial set $S\cup \left\{u\right\}$ and select the combinatorial set with the maximum utility. Finally, utilize the result as the newly selected participant set for the next iteration. In this paper, the utility of user combinatorial set is defined as the expectation of sensing area covered by $S\cup \left\{u\right\}$ in all sensing cycles, and its calculation formula is as follows:

(11) $$Utility\left(S\cup \left\{u\right\}\right)=\sum_{0\leq i<N}\sum_{a\in A}Q_{i,a}\left(S\cup \left\{u\right\}\right),$$

The task assignment mechanism based on user utility calculates the utility of each combination set, selects the combination set with the maximum utility, and continues the next iteration until the stop criterion is met. After obtaining the set of combinations with maximum utility $\left(S\cup \left\{u\right\}\right)$, the algorithm computes a covering probability vector, where each element of the vector is the probability of being covered by the combinatorial set $\left(S\cup \left\{u\right\}\right)$ with a preset percentage (R) of the perceived region in the corresponding perceptual cycle. The following formula gives the probability calculation formula in the i-th perception cycle.

(12) $$\begin{array}{cc} {COV}_{i}\left(S\cup \left\{u\right\}\right) & =\sum_{A_{c}\in A}^{\left|A_{c}\right|\geq \tau }\prod_{\forall a\in A_{c}}Q_{i,a}\left(S\cup \left\{u\right\}\right)\\ & *\prod_{\forall a^{\prime }\in A\setminus A_{c}}\left(1-Q_{i,a^{\prime }}\left(S\cup \left\{u\right\}\right)\right), \end{array}$$

where $\tau =\left|A\right|*R$ indicates the number of the minimum sensing areas that should be covered in each sensing cycle, and $A_{c}\left(\left|A_{c}\right|\geq \tau \right)$ is a subset of the sensing area set A, indicating the combination of sensing areas that should be covered.

After obtaining the probability vector calculated based on the aggregation of the maximum utility user group, the algorithm identifies the minimum probability value within the vector. It then compares this minimum probability value with the given stop threshold. If the minimum probability is greater than or equal to the stop threshold, the combination set returned will be considered as the final user set selected by the algorithm. On the other hand, if the minimum probability falls below the stop threshold, the algorithm will use the combination set as the selected participant and initiate the next iteration. To summarize, the algorithm iteratively repeats the process until it finds a combination set with a minimum probability value greater than or equal to the stop threshold. This set will then be used as the final selected user set.

Given the number of users |U|, in the worst case, the proposed algorithm UBTA runs |U|∗(|U|+1)/2 iterations (i.e., all users are selected) to obtain the result, and in the best case, the algorithm runs only |U| iterations (i.e., one user is selected) to obtain the result.

## 6. Performance Evaluation

### 6.1. Experiment Settings

In this section, we evaluate the proposed UBTA algorithm, first design the greedy algorithm for comparison, then introduce the experimental settings, and finally give the detailed experimental results between the UBTA algorithm and the comparison method.

For the task assignment problem in this chapter, we designed a greedy algorithm (GA), whose basic idea is to select from all unselected vehicle users in each iteration the user that covers the most perceived area in the history of call records until the set probability threshold is reached and the cycle stops. We will compare the UBTA algorithm with the GA algorithm in subsequent experiments.

This paper conducts an analysis and study of passenger flow patterns at various passenger hotspots during different time periods by mining GPS historical data from taxis. The dataset used comprises GPS historical data from 4000 taxis in Shanghai, collected over the course of one month. To account for the distinct travel patterns of residents on working days and weekends, the dataset is divided into two subsets: one for working days and the other for weekends. With the massive GPS historical data available in these subsets, this study quantifies the number of passenger-carrying events occurring at different time periods within the passenger hot spot areas. To predict passenger flow in various regions, the Gaussian process regression algorithm is employed. Ultimately, these predictions aim to enhance the efficiency of taxi passenger transportation.

The setup for our study is as follows: we set the number of perception areas, denoted as $\left|A\right|$, to 45. The project involves 10 cycles, each spanning from 8:00 to 18:00, with each cycle lasting for 1 h. The sensing duration required for each task is δ=3 min. Additionally, we assume that the noise added to the user dwell time data follows the standard Laplacian distribution, with a privacy budget parameter ϵ=1. In this study, we conducted a two-week experiment using a computer-generated simulation dataset. The data from the previous week served as the training dataset, used for task assignment. On the other hand, the data from the following week acted as the test dataset, simulating the spatio-temporal coverage of the selected participants. The experiment dataset comprises call records and residence time data of 1000 vehicle users in each space-time unit. It includes user ID, signal tower ID, the number of calls in each space–time unit, the real residence time of users, and the residence time after adding noise. The completion of the task by vehicle users depends on two factors: whether they make a call in a specific space–time unit and whether their stay time is longer than the sensing duration required for the task.

### 6.2. Experimental Results

Table 1 shows the comparison of the number of vehicle users who are selected by the UBTA algorithm and the GA algorithm. Evidently, the performance of the UBTA algorithm is better than the GA algorithm. When the preset coverage value is 95%, the number of vehicle users recruited by UBTA is 10.11–54.80% less than that recruited by GA. When the coverage default is 85%, the number of vehicle users recruited by UBTA is 7.94–56.89% less than the number of vehicle users recruited by GA.

It is worth noting that, when the coverage default is 50%, there is little difference between the number of vehicle users recruited by the UBTA algorithm and the GA algorithm. Therefore, we compared the vehicle users selected by the two algorithms when the coverage preset was 95%, 85%, and 50%, respectively, and calculated the proportion of common users recruited by the two algorithms (i.e., the proportion of vehicle users in the intersection of recruitment results). As shown in Table 2, when the preset coverage value is 50%, the average proportion of common users recruited by the algorithm is 77.43%, much higher than 54.61% and 40.82% when the preset coverage value is 95% and 85%, which means that most users recruited by the GA algorithm in this case are also in the set of users recruited by the UBTA algorithm. We call the users recruited by the UBTA algorithm high-quality users (that is, those users can reach the preset coverage rate in a small number); therefore, the GA algorithm recruits high-quality users in the majority of selected users when the preset coverage rate is 50%, which is also the reason why the number of users recruited by the GA algorithm is close to the UBTA algorithm at this time.

**Figure 3:**
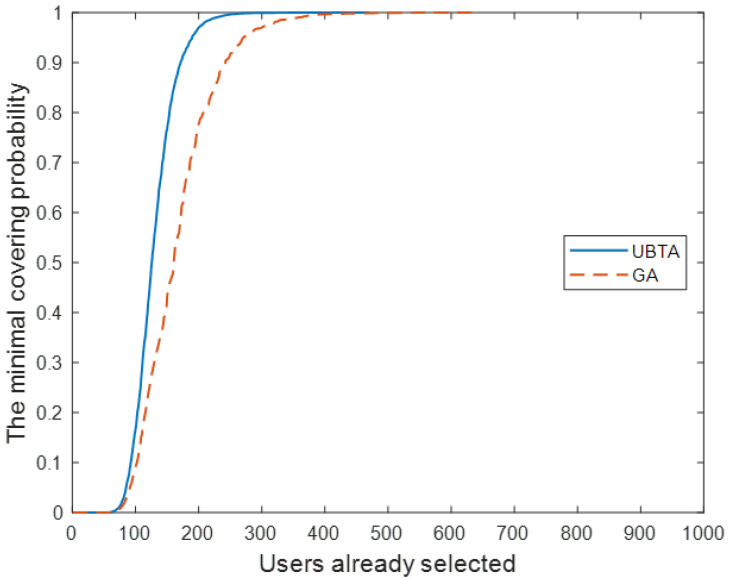
Participant selection process.

First, Figure 3 shows how the minimum coverage probability in all sensing cycles varies with the number of mobile users selected. Clearly, the minimum coverage probability of UBTA grows faster, and its curve converges to a pre-set threshold with a smaller number of mobile users.

**Figure 4:**
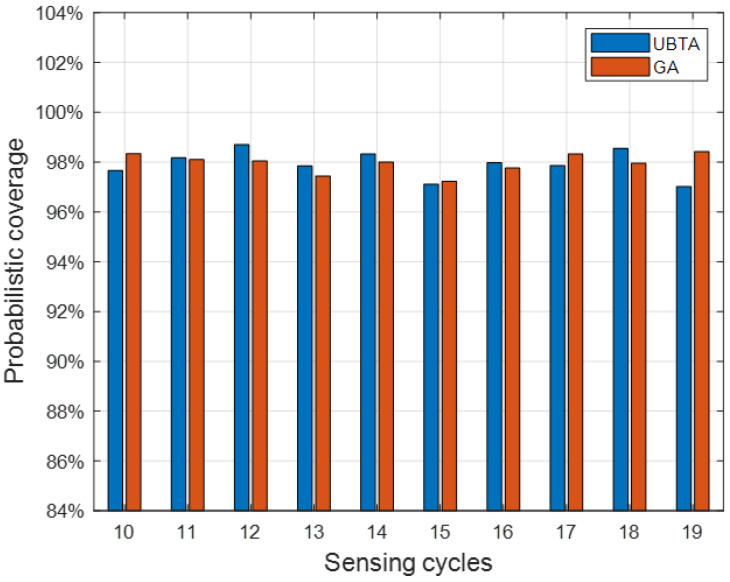
Average coverage of sensing areas: R = 95%.

Next, we set the coverage default to 95% and compared the process of selecting vehicle users by UBTA and GA. Figure 4 shows how the minimum coverage probability varies with the number of selected vehicle users in all sensing cycles. Clearly, the minimum coverage probability of UBTA grows faster and its curve converges to a pre-set threshold with a smaller number of vehicle users.

**Figure 5:**
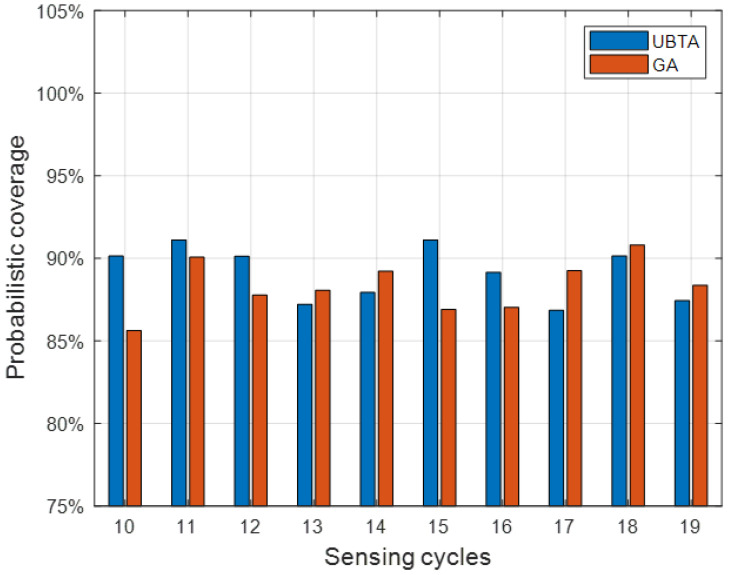
Average coverage of sensing areas: R = 85%.

Finally, we test the effectiveness of the two algorithms, that is, test data are used to test whether the participants recruited by the algorithm can reach the pre-set coverage. Figure 5 shows the average percentage of the sensing area covered by the recruited participants in each sensing cycle. As can be seen from Figure 3 and Figure 4, for the UBTA algorithm and the GA algorithm, the coverage of perception area in each sensing cycle has reached the pre-set threshold of 95% and 85%, thus demonstrating the effectiveness of the two algorithms. Overall, the UBTA algorithm recruited fewer vehicle users to meet pre-set coverage criteria, thereby reducing perceived costs.

## 7. Conclusions

In this paper, we address the task allocation challenge in vehicular crowdsensing through an opportunistic approach based on the real-world scenario. We propose a user utility-based task allocation algorithm that involves several key steps. Firstly, we predict the mobility of users to understand their movement patterns. Next, we calculate the utility of various user combinations to assess their effectiveness in completing tasks. Additionally, we measure the probability of sensing task coverage for each user combination. Based on these calculations, we determine the set of participants with the minimum number of users required for optimal task allocation. To protect user privacy, we employ differential privacy technology. Users upload their residence time data with Laplace noise, ensuring that their sensitive information remains secure. The platform then analyzes user mobility by solving the probability problem of continuous random variables, utilizing the fuzzy residence time. The proposed algorithm is further analyzed theoretically, including time complexity and efforts to reduce the computational complexity of the coverage probability vector. Finally, we conduct experimental evaluations to demonstrate the effectiveness of the proposed algorithm. 

## Figures and Tables

**Table 1:** The number of vehicle users selected by UBTA and GA.

Sensing Program	R=95%		R=85%		R=50%	
	UBTA	GA	UBTA	GA	UBTA	GA
1	113	250	58	63	20	21
2	471	644	228	321	71	72
3	456	675	222	398	69	72
4	502	620	235	298	70	72
5	449	696	216	501	68	73
6	711	791	316	458	93	94
Average	450.33	612.67	212.5	339.83	65.17	67.33

**Table 2:** Percentage of shared vehicle users among UBTA and GA.

Sensing Program	R=95%	R=85%	R=50%
1	44.65%	32.00%	79.85%
2	58.85%	47.04%	77.78%
3	55.41%	39.45%	75.42%
4	66.61%	48.99%	73.61%
5	52.30%	30.74%	80.23%
6	49.86%	46.72%	77.66%
Average	54.61%	40.82%	77.43%

## Data Availability

No new data were created or analyzed in this study. Data sharing is not applicable to this article. No public involvement in any aspect of this research.
